# Metabolome and Transcriptome Analyses Reveal the Correlation Between Fructan Changes and Phytohormone Regulation During Tuber Sprouting of *Helianthus tuberosus* L. †

**DOI:** 10.3390/ijms26051864

**Published:** 2025-02-21

**Authors:** Ya Wen, Zhenjie Zhou, Xiaozhu Guo, Juan Li, Gui Wang, Xuemei Sun

**Affiliations:** 1 Academy of Agriculture & Forestry, Qinghai University, Xining 810016, China; wenya103401@163.com (Y.W.); shy1998zzj@163.com (Z.Z.); guoxzh2024@lzu.edu.cn (X.G.); raureen@foxmail.com (J.L.); wanggui199710@163.com (G.W.); 2 Laboratory for Research and Utilization of Germplasm Resources in Qinghai Tibet Plateau, Qinghai University, Xining 810003, China

**Keywords:** jerusalem artichoke, metabolic regulation, fructan, phytohormone, omics analysis

## Abstract

Jerusalem artichoke (*Helianthus tuberosus* L.) reproduces asexually through tubers, which are rich in fructan-type carbohydrates and serve as the primary processing organ. Plant hormones may regulate the sprouting process of tubers, but the changes in fructans and their regulatory mechanisms in relation to hormones remain unclear. This study utilized two varieties of Jerusalem artichoke, “Qingyu No.1” and “Qingyu No.3”, to analyze the changes in the proportion of carbohydrates (especially fructans) in total sugars during the sprouting process. Metabolomic and transcriptomic analyses were conducted at three selected sprouting stages. The results indicated that during tuber sprouting, carbohydrates such as fructans play a regulatory role through conversion activities. Multi-omics analysis revealed that jasmonic acid and salicylic acid promote Jerusalem artichoke sprouting through metabolism and are involved in the plant hormone signal transduction process. Differentially expressed genes related to hormone signaling were screened and divided into three groups based on expression levels. It was found that the proportion of carbohydrates is highly correlated with the expression of hormone-related genes in the sprouts, suggesting that plant hormones may regulate carbohydrate metabolism during the sprouting of Jerusalem artichoke tubers. In conclusion, these results preliminarily elucidate the regulatory mechanisms of plant hormones in Jerusalem artichoke tuber sprouting, aiming to provide a theoretical basis for the production and processing of Jerusalem artichoke.

## 1. Introduction

Jerusalem artichoke (*Helianthus tuberosus* L.), a sunflower in the asteraceae family, is a perennial herb. Jerusalem artichoke is native to North America and was introduced to China from Europe. It is now mostly grown in the northwest of China. Jerusalem artichoke is a plant rich in various chemical components, including inulin, a natural macromolecular linear polymer, which accounts for about 80% of the dry weight of the tuber and has health-care functions of regulating blood sugar and intestinal flora; In addition, Jerusalem artichoke also contains fatty acids, terpenoids, phenolic acids, and other compounds, which have different biological activities. These rich chemical components give Jerusalem artichoke broad development and application prospects in the fields of food, medicine, and the chemical industry [1]. Tuber germination is the initial stage of the life process of Jerusalem artichoke, and the regulation activities in this stage have an important effect on its later growth and development. Jerusalem artichoke uses fructose-based inulin as the storage polysaccharide, rather than the sucrose-based starch more commonly used by plant species, with the tuber being the main site of inulin accumulation [2]. Inulin is an example of fructose-based polysaccharide dietary fibers known as fructans, which can be used to regulate sugar transportation, storage, and usage [3]. Secondly, in addition to fructan, it also contains carbohydrates such as fructose, glucose, and sucrose, which can be used as precursor substances for the synthesis of fructan and are also the product of the decomposition of fructan.

Studies have shown that oligosaccharides have physiological regulatory effects on plants, with oligomers being particularly effective in promoting plant growth and enhancing cold tolerance [4]. Initially, the Jerusalem artichoke was primarily used as a food source, but later, it was also employed for other purposes. Bio-extraction methods were employed to process the tubers, increasing the biomass value of inulin, fructan, and other components [5]. Research has found that after sprouting, the starch content in potatoes, which is composed of glucose units, decreases while the reducing sugar content increases [6]. Jerusalem artichoke fructan is a polyfructan linked by β-1,2-glycosidic bonds, with a glucose residue at the end, having a relative molecular mass of 3500–5500 and a degree of polymerization (DP) of 2–60 [7]. During the reproductive period of Jerusalem artichokes, fructan is involved in the conversion and distribution of carbohydrates, and its metabolic regulation plays a crucial role in plant growth [8].

During asexual reproduction via tubers, the sprouting period significantly impacts subsequent growth and development [9]. Seed germination is regulated by mechanisms including plant hormones [10]. Tuber dormancy and sprouting are critical physiological stages affecting growth and yield, influenced by both genetic background and environmental factors. As regulatory small molecules, plant endogenous hormones control tuber sprouting and dormancy, exerting significant physiological effects at extremely low concentrations (10^−9^–10^−6^ mol/L) and playing a key role in plant growth and development by regulating gene expression [11].

Jerusalem artichoke tubers contain endogenous hormones, and their growth and development are closely related to changes in hormone levels. Yu Zhang measured the content of endogenous hormones in tubers during storage using the ELISA method and found that the levels of gibberellin and indoleacetic acid significantly increased after dormancy was broken, suggesting that an increase in growth-promoting endogenous hormones can break dormancy [12]. Research using potatoes as experimental material showed that endogenous auxin increases during the early stages of tuber development and then decreases [13]. Genes related to auxin response factors are expressed in the bud tips, with expression levels significantly increasing during the early stages of sprouting [14]. Zubko found that potato tubers with higher cytokinin content have shorter dormancy periods and stronger sprouting abilities [15]. In sweet potato tubers, various endogenous hormones, such as cytokinin, auxin, and gibberellin, work together during the sprouting period [16]. Analysis of endogenous hormones before and after sprouting in creamer potatoes revealed an increase in gibberellin content and cytokinin activity [17].

The sprouting of Jerusalem artichoke tubers is crucial for their processing, utilization, and reproduction. The tubers are rich in fructan-type carbohydrates, which hold significant importance in processing and utilization.

Currently, the metabolic characteristics of fructans during tuber sprouting, the relationship between hormone metabolism and tuber sprouting, and the synergistic effects between hormone metabolism and fructan metabolism remain unclear. Therefore, this study utilized two varieties: “Qingyu No.1” (QY1), an early-maturing variety with purple-red skin and white flesh. “Qingyu No.3” (QY3), a late-maturing variety with white skin, large tubers, and fewer fibrous roots, bred by the Jerusalem Artichoke Research and Development Center of the College of Agriculture and Forestry Sciences at Qinghai University. The changes in carbohydrates during the sprouting process were measured to explore the allocation patterns of energy substances. Combined with the changes in plant hormone content within the sprouts, the regulatory role of hormones in Jerusalem artichoke sprouting was investigated. Additionally, based on multi-omics (metabolomics and transcriptomics) technologies, differentially expressed genes at different sprouting stages were analyzed to preliminarily elucidate the regulatory mechanisms of plant hormones in Jerusalem artichoke tuber sprouting. This study aims to provide theoretical guidance for production techniques in Jerusalem artichoke cultivation.

## 2. Results

### 2.1. Dynamics of Carbohydrate Changes During Sprouting

**Figure 1:**
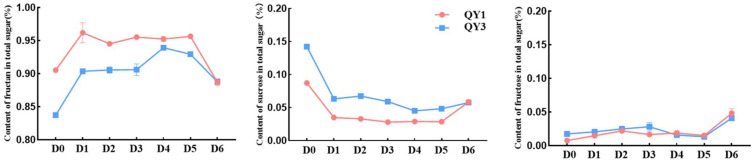
Proportion of carbohydrate in total sugar of Jerusalem artichoke tubers during sprouting.

The proportion of fructan in total sugar showed an initial upward and then downward trend throughout the sprouting period. The proportion of tuber fructan decreased by approximately 1.88% overall in QY1, while it increased by 5.03% in QY3. The proportion of sucrose of tubers decreased and then gradually increased, while the fructose showed an upward trend (Figure 1). During the period from the sprouting to the expansion of buds and the beginning of the loosening of scales, the proportion of sucrose rapidly decreased and was released as fructose while being synthesized into fructan. The proportion of carbohydrate was stable from sprouting to true leaf formation. Fructan was decomposed into free sugars during the late stage of sprouting, before and after the formation of true leaves. It was inferred that during bud formation, Jerusalem artichoke tubers synthesize fructan to prepare for subsequent growth consumption; before and after the formation of true leaves, fructans began to decompose to provide accumulated energy for the growth of Jerusalem artichoke. Overall, Jerusalem artichoke stabilizes the plant sprouting process through the coordinated metabolism of fructans and free sugars.

The proportion of carbohydrate of the tubers varied significantly in the D1 and D4 periods. Therefore, three QY1 tissue samples were selected from the D0, D1, and D4 periods, namely, tubers from D0 stage and buds from D1 and D4 stages, for metabolite extraction, identification, and analysis of differential metabolites and genes within the phytohormone signaling pathway.

### 2.2. Analysis of Differential Metabolites

PCA and OPLS-DA [18,19] showed a large difference between the sample groups but a slight difference within the groups (Figure S1). The overlap display analysis of the total ion flow plots by mass spectrometry detection of various QC samples revealed that the total ion flow curves for metabolite detection had a high overlap (Figure S2). The instrument’s high stability ensures data reproducibility and reliability.

**Figure 2:**
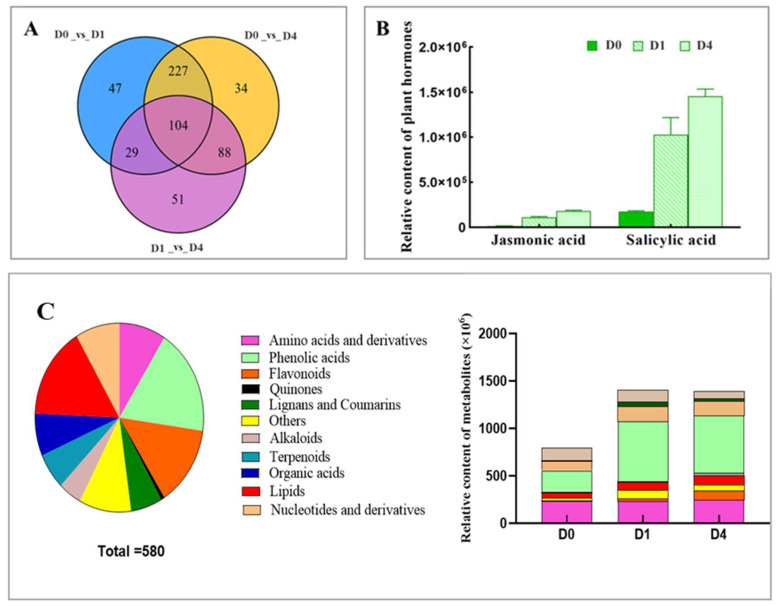
Metabolite analysis of Jerusalem artichoke tissue during sprouting. Note: (**A**): Venn diagram of differential metabolite; Each circle represents a comparison group. Overlapping areas show the number of shared differential metabolites, while non-overlapping areas indicate unique differential metabolites. (**B**): relative content of plant hormone-related differential metabolite; (**C**): Pie chart showing differential metabolite classification and the difference in the relative content of each metabolite class.

Metabolites with fold change ≥ 2, fold change ≤ 0.5, and VIP ≥ 1 were selected as significantly different metabolites. Of the 580 different metabolites screened, the content of 104 differed significantly in all three stages, and 308 differed significantly before and during the sprouting and did not differ significantly between D1 and D4 (Figure 2A).

All differential metabolites were analyzed according to the categories of substance-level classification (Figure 2C). The top three categories with the highest number of metabolites were phenolic acids, lipids, and flavonoids, containing 106, 92, and 80 differential metabolites, respectively. Quinone had the least number of metabolites, with only four differential metabolites, whose relative content was also the lowest among all differential metabolites, with an increasing trend from 0.04 × 10^6^ to 0.61 × 10^6^ in D0, D1, and D4. Phenolic acid had the highest relative content among all differential metabolites, with approximately 631.70 × 10^6^ in D1.

Salicylic acid and jasmonic acid, two of the eight phytohormones, differed during sprouting. The relative salicylic acid content differed before sprouting and was upregulated during sprouting in D1 and D4, and the differences were not significant between D1 and D4. Similar results were obtained for the jasmonic acid content. Salicylic acid and jasmonic acid are jointly annotated to three pathways, of which map01100 is a metabolic pathway, map01110 is secondary metabolite biosynthesis, and map04075 is the phytohormone signaling pathway (Table S1). Subsequent analyses were conducted to determine relevant DEGs within this pathway.

Salicylic acid was found to be more abundant than jasmonic acid in the buds from the beginning of tuber sprouting, with the relative contents of salicylic acid and jasmonic acid increasing from 1.72 × 10^5^ to 14.53 × 10^5^ and 0.13 × 10^5^ to 1.79 × 10^5^, respectively (Figure 2B).

### 2.3. Analysis of DEGs

In total, 58.90 Gb of clean data were generated from 9 transcriptomic samples, with Q20 base percentage > 96%, Q30 base percentage > 91%, and GC content > 44% (Table S2). The function of several unigenes remains unknown due to the lack of Jerusalem artichoke genome information and other sequencing data. Three databases were used for unigene annotations: KOG [20], GO [21], and KEGG [22].

The most frequent KOG annotation, aside from “General function prediction only”, was “Signal transduction mechanisms”, indicating the importance of phytohormones in the sprouting of Jerusalem artichoke (Figure S3). The most enriched categories in the GO function database for “Biological Process” were “cellular process” and “metabolic process”, for “Cellular Component” were “cell” and “cell part”, and for “Molecular Function” were “binding” and “catalytic activity” (Figure S4). The groups “Metabolism” and “Genetic Information Processing” had the highest number of KEGG database terms. The largest category was “Ribosome” in “Genetic Information Processing” (Figure S5).

Four pathways were annotated in the “Environmental Information Processing” category, with D0/D1 annotating 599 DEGs in the “phytohormone signaling system”, D0/D4 annotating 642 DEGs, and D1/D4 annotating 149 DEGs (Figure S5). The results suggest that phytohormone signaling is active in Jerusalem artichoke before and after sprouting; however, the differences in related genes do not change significantly during sprouting.

Around 33,597 DEGs were identified and compared; 200 were differentially expressed at three stages, 32,535 differed in expression only before and after sprouting, and there was no difference in D1 and D4. The enrichment of phytohormone-related differential genes in each period of Jerusalem artichoke sprouting was examined using KEGG enrichment analysis. Compared with D0, the D1 and D4 periods were enriched in the phytohormone signaling pathway (map04075). Jasmonic acid and salicylic acid are both jointly annotated to this pathway in the analysis of differential metabolites. We screened all genes that could be annotated to this pathway and classified the genes with |coefficient| > 0.99 into phytohormone types (auxin, cytokinin, gibberellin, abscisic acid, ethylene, brassinolide, jasmonic acid, salicylic acid) based on the KOG database.

Phytohormones act as signaling small molecules through the phytohormone signaling pathway during plant growth and development [23]. PCA was used to analyze the expression data of all genes related to phytohormone signaling, which revealed different expression patterns of different hormones (Figure S6A). We annotated 92 phytohormone-related differential genes and constructed a differential gene co-expression network. Different hormones occupy different positions in the network, with brassinolide, auxin, and gibberellin occupying the most critical positions (Figure S6B).

**Figure 3:**
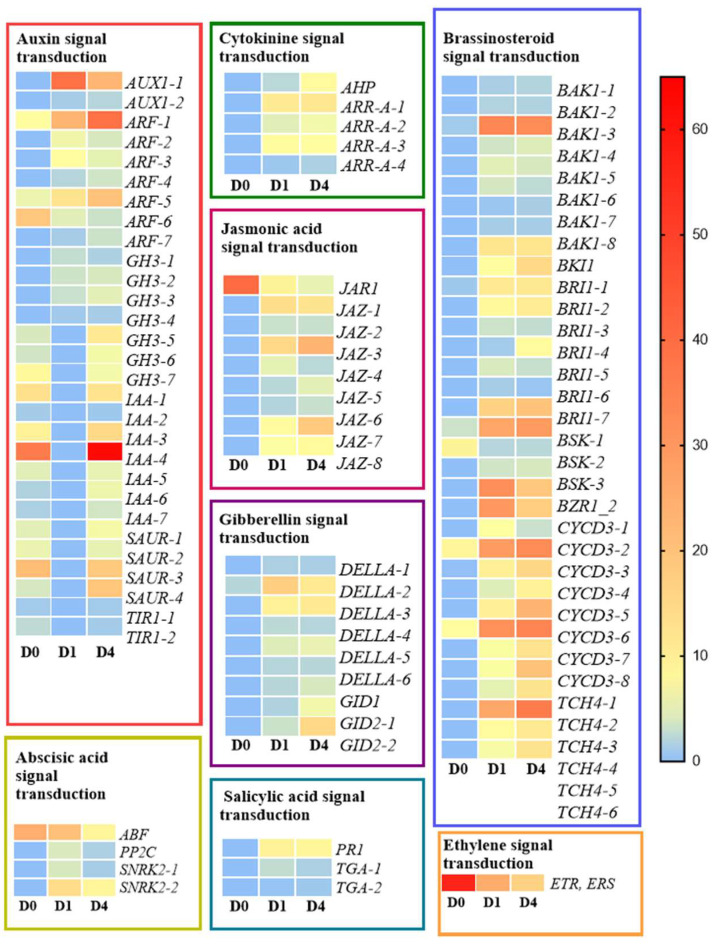
Phytohormone-related differential genes in tissue of Jerusalem artichoke during sprouting. Note: The relative expression levels of differential genes.

In eight different plant hormone signal transduction pathways, unigenes were annotated to all types of plant hormones. Among them, the number of differentially expressed genes related to auxin and brassinosteroids was the highest, with 29 and 34 genes, respectively, while the number of differentially expressed genes related to ethylene was the lowest, with only one gene (Figure S7). Overall, the results indicate that the influence of plant hormones during Jerusalem artichoke sprouting is comprehensive and extensive. To further explore the expression patterns of these genes, we used a heatmap to display the expression levels of all unigenes annotated to plant hormone signal transduction (Figure 3). The expression levels of most differentially expressed genes related to plant hormone signal transduction increased across the three periods.

### 2.4. Combined Analysis of Hormone-Related Differential Metabolites and Differential Genes

Phytohormone signal transduction pathways were enriched in the DEGs during sprouting, demonstrating the complexity of phytohormone responses to the growth and development of Jerusalem artichokes. All genes related to the signaling of the eight phytohormones were studied to elucidate the transcriptional dynamics of phytohormones regulating Jerusalem artichokes during early sprouting (Figure S8).

The results showed that the DEGs were distributed between all eight hormones; however, the contents of jasmonic acid and salicylic acid among the corresponding metabolites differed significantly. Correlation analysis of the two phytohormones and the related differentially expressed genes in the corresponding pathways showed that each gene is significantly correlated with the metabolites (*p* < 0.01). *JAR1* continued to be downregulated during the sprouting process, having a negative correlation with endogenous hormones. It was believed that the increase in the content of endogenous hormones has a significant inducing effect on gene expression in the signal transduction pathway (Figure S9).

### 2.5. Correlation Analysis of Carbohydrate Dynamics and Phytohormone-Related DEGs

DEG expression analysis revealed significant negative correlations for auxin (r = −0.97, *p* < 0.001), brassinolide (r = −0.96, *p* < 0.001), gibberellin (r = −0.94, *p* < 0.001), and ethylene. Cytokinin (r = 0.91, *p* < 0.001) and gibberellin were significantly positively correlated. Salicylic acid (r = −0.77, *p* = 0.01–0.05) and ethylene were negatively correlated. Jasmonic acid (r = 0.80, *p* < 0.01) and cytokinin were positively correlated. Therefore, we classified the phytohormone-related DEGs into three categories: ethylene, abscisic acid (|r| < 0.07, *p* > 0.05), and auxin, cytokinin, brassinolide, gibberellin, jasmonic acid, and salicylic acid.

**Figure 4:**
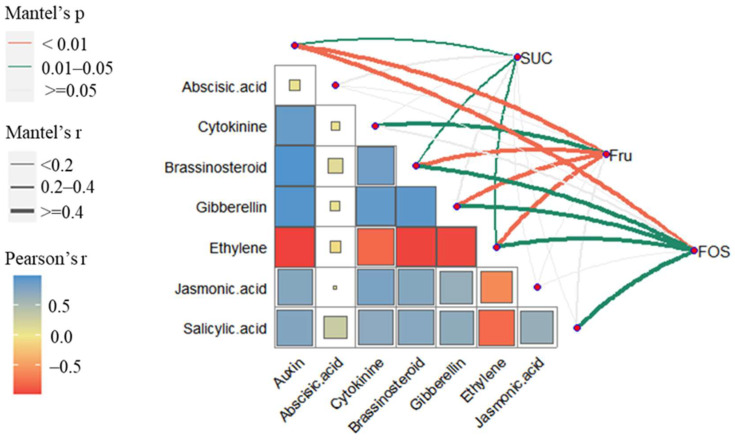
Correlation between carbohydrate dynamics and phytohormone-related differentially expressed genes across periods. Note: A box inside the heatmap indicates a correlation; blue and red represent Pearson’s r value; links with *p*-value and |r| value show the correlation between carbohydrates and phytohormones. The width of the edges indicates the discrete Mantel’s r, and the red and green colors indicate Mantel’s p.

The Mantel test for the proportion of carbohydrate and phytohormone gene expression showed that changes in the expression of genes related to ethylene in the “ethyl-ene” category were correlated with the proportion of sucrose and fructan during sprouting (*p* = 0.01–0.05) and significantly correlated with fructose (*p* < 0.01). Abscisic acid-related gene expression in the second category was not related to carbohydrates (*p* > 0.05). The gene expression of hormones in the third category, except for jasmonic acid (*p* > 0.05), was related to carbohydrates, and all but salicylic acid (*p* > 0.05) were more correlated with fructose. Only auxin was highly significantly correlated with fructan (*p* = 0.008). Auxin (*p* = 0.001), brassinolide (*p* = 0.001), gibberellin (*p* = 0.002), and ethylene (*p* = 0.002) were significantly correlated with fructose (Figure 4).

**Figure 5:**
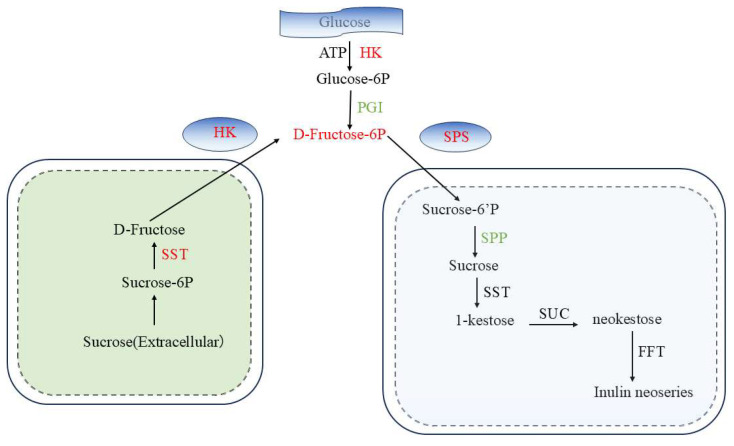
Fructan regulatory network in Jerusalem artichoke.

Secondly, fructan synthesis first generates glucan-6p from glucose under the action of HK, then D-fructose-6p under the action of PGI, and finally, fructan under the action of SPS, SST, and SUC (Figure 5).

Studies have found that the metabolism of fructan is related to plant growth regulators. We screened hormone-related genes during the germination process of Jerusalem artichoke and conducted CCA analysis of sugar-related metabolites. We found that some hormone-related genes were also related to sugar metabolites. *ARF*, *CH3*, *CYCD3*, *TCH4*, and other genes are strongly correlated with metabolites such as Glucose-1-phosphate, D-fructose-6-phosphate, and D-glucose-6-phosphate, which are positively regulated, while nystose and planteose are negatively regulated (Figure S10).

According to the correlation of gene expression, they were divided into different types, and the classification results were consistent with the functional types of each hormone. Except for abscisic acid, the proportion of carbohydrates to total sugars in each group was similar to the expression of plant hormone genes. We suggest that the proportion of fructose as a synthetic precursor and fructan hydrolysate is significantly influenced by plant hormones, mainly related to hormones regulating plant germination and growth. Auxin-related genes are closely related to fructan metabolism.

## 3. Discussion

### 3.1. Dynamics of Fructan Changes

Jerusalem artichokes reproduce asexually through tubers rich in fructans [24]. Research has shown that SST sucrose: sucrose-fructosyltransferase is a key gene regulating fructan biosynthesis and is responsible for the accumulation of fructans in Jerusalem artichokes together with FFT fructan: fructan-fructosyltransferase [25]. During the period from germination to the loosening of the scales, fructan synthesis is active, the proportion of sucrose decreases, and the proportion of fructan increases [26]. During the period from germination to the formation of true leaves, the proportion of fructan, sucrose, and fructose in the tubers remained stable overall; Before and after the formation of true leaves, the proportion of fructan decreased, while the proportion of sucrose and fructose increased, indicating that fructan was decomposed into free sugars to provide energy for the growth of Jerusalem artichokes. In different cultivation environments, the content of fructan increases after the germination of Jerusalem artichoke tubers, while the content of sucrose and fructose decreases [27], which is consistent with the results of this study.

Fructan metabolism is affected by plant growth regulators, and its molecular size, structure, and localization vary depending on the regulator [8]. In the future, based on the changes in carbohydrate ratio, we can study the metabolic enzymes and their activities during germination, analyze the metabolic characteristics of fructans, and explore the interactions between differentially expressed plant hormone genes and enzymes in the carbohydrate metabolism network.

### 3.2. Phytohormone Regulation Network

We analyzed the metabolome and transcriptome of Jerusalem artichoke at D0 before germination, stage 1 (D1), and stage 4 (D4). Among the eight plant hormones, the relative content of salicylic acid and jasmonic acid increased significantly during germination, and the content of salicylic acid was higher than that of jasmonic acid, both showing an upward trend. Existing research has focused on the resistance effects of salicylic acid and jasmonic acid, which are the core of hormonal regulation in the immune system [28]. These two hormones can interact with each other to enhance plant resistance. Salicylic acid affects the activity of H+-ATPase in the plasma membrane of potatoes, while jasmonic acid can enhance this activity [29]. Jerusalem artichokes have strong stress resistance, and research has shown that jasmonic acid can regulate their life processes, promote germination, and induce the formation of tubers [30]. Therefore, the significant increase in the content of salicylic acid and jasmonic acid during germination is of research significance.

This study reveals that the contents of salicylic acid and jasmonic acid significantly increase before and after the sprouting of Jerusalem artichoke, sharing the same signal transduction pathway. The significant correlation between hormone content and differential gene expression indicates that these two hormones promote sprouting through metabolic regulation and participate in signal transduction. Linda’s study found that the content of jasmonic acid in potato tubers increases fourfold during sprouting, consistent with our results [31].

The salicylic acid signaling pathway is well-defined, transmitting signals through binding proteins (SABPs) to activate transcription factors (such as NPR1, TGA, and WRKY), thereby inducing PR gene expression [32]. In this study, the increase in salicylic acid content after sprouting led to the downregulation of the TGA gene and reduced expression of the PR1 gene. In the jasmonic acid signaling pathway, COI1 and JAZ proteins are key components. JAILE binds to the SCFCOI1 complex, promoting JAZ degradation and releasing transcription factors (such as MYC2) to activate downstream genes [33]. The increase in jasmonic acid content before and after sprouting induced the upregulation of the JAZ gene, while other genes showed no significant changes.

Genes related to all eight phytohormones are involved in the sprouting of Jerusalem artichoke, with auxin, brassinolide, and gibberellin having the highest number of differentially expressed genes.

Auxin is a key signaling molecule regulating plant growth and development, functioning through biosynthesis, catabolism, and signal transduction pathways [34]. The typical signaling pathway includes core components such as *GH3*, *SAUR*, *AUX/IAA*, and *ARF* [35]. Changes in auxin concentration rapidly affect the expression of early response genes [36], and auxin enters cells via AUX1, where it is perceived by SCFTIR1/AFB and TIR1 [37]. This study found coexisting upregulation and downregulation of gene expression during the sprouting process.

Brassinolide (BR) is a sterol-like phytohormone that regulates various physiological processes, including seed sprouting [38,39]. BRI1 is a BR receptor that detects signals and activates BSK kinase to initiate signal transduction [40], during which BAK1 acts as a co-receptor and BZR1/2 as activated transcription factors, promoting gene expression related to cell division and growth [41,42]. In this study, the expression of *CYCD3*, which promotes cell division [43], and *TCH4*, which promotes cell elongation, increased monotonously during sprouting [44].

The GA-GID1-DELLA signaling pathway is a well-defined gibberellin signaling pathway [45]. In *Arabidopsis thaliana*, gibberellin receptor GID1 and its binding protein undergo conformational changes and then combine with DELLA protein to form a trimer, which binds to the SCF complex and ubiquitinates the DELLA protein, ultimately realizing the regulation of gibberellin on plant growth [46]. *GID1* and *GID2* in the buds were monotonously upregulated during sprouting in this study, and *DELLA* gene expression was the highest in the buds during the D1 period, demonstrating a dynamic of increase and decrease. We hypothesize that these genes interact with other phytohormone-related genes through upregulation to promote tuber sprouting.

These three types of phytohormone levels did not differ during the sprouting of Jerusalem artichoke and require further investigation. We suspect the hormone content is too low to reach the detection threshold. These hormones regulate related gene expression changes by upregulating or downregulating them within the pathway.

### 3.3. Correlation Analysis of Carbohydrates and Phytohormone-Related DEGs

Plant hormones coordinate during growth, development, and senescence, each having a distinct regulatory mechanism [47]. Based on different phytohormone gene expression correlations, three categories were identified during the sprouting of Jerusalem artichoke. Ethylene is mostly active in the mature stage of plant fruit development [48]. Abscisic acid regulates seed dormancy and sprouting [49]. In addition, ethylene and abscisic acid metabolism and reactive oxygen species (ROS) homeostasis coordinate and interoperate in the early stage to release seed dormancy [50]. In this study, we segregated ethylene and abscisic acid based on their Pearson coefficients. Auxin, gibberellin, and brassinolide interact to regulate plant growth and development [51,52]. Kolachevskaya used isolated potatoes to demonstrate crosstalk between auxin and cytokinin signaling pathways [53]. Salopek-Sondi used cabbage-type rapeseed to demonstrate that jasmonic acid and salicylic acid are stress hormones that affect the gene transcription involved in the reversible binding process of auxin [54]. In the correlation analysis of this study, the other six hormones were classified into a third category, and they were positively correlated with each other and negatively or not significantly correlated with ethylene and abscisic acid, suggesting the regulatory mechanism of the hormones.

The proportion of carbohydrate in total sugar was correlated with phytohormone gene expression, except for abscisic acid; the correlation coefficient was higher for fructose. Fructose is an important carbohydrate used as a precursor for fructan synthesis in Jerusalem artichoke, which regulates plant growth and development through metabolism [55]. Bingchao Wu and Linkai Huang found that phytohormones influence pearl millet seed sprouting and carbohydrate metabolism, which is consistent with our results [56]. Therefore, it was inferred that phytohormones regulate fructose-based carbohydrates during the sprouting of Jerusalem artichoke tubers. Phytohormones that regulate plant sprouting and growth significantly influence the proportion of fructose as a synthesis precursor and fructose hydrolysate. Overall, these findings provided a basis for establishing plant carbohydrate and phytohormone co-expression networks. Secondly, on the basis of this study, the function of jasmonic acid and salicylic acid in the germination of Jerusalem artichoke tubers was subsequently verified, the function of selected plant hormone-related differential genes was verified, and the activity of upstream genes of each pathway was studied. The plant hormones in the process of tuberous germination of Jerusalem artichoke were extracted and determined with high accuracy. It can further elucidate the plant hormone gene network and provide a theoretical basis for studying the regulatory mechanism of Jerusalem artichoke tuber during germination.

## 4. Materials and Methods

### 4.1. Materials

**Figure 6:**
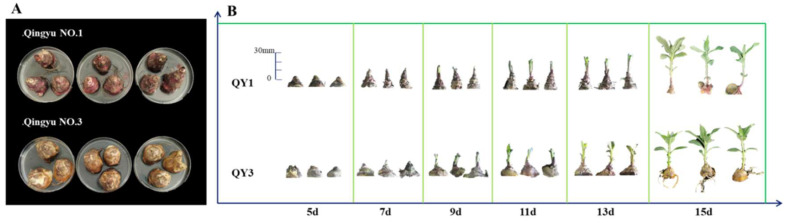
Jerusalem artichoke. Note: (**A**): Sprouting test; (**B**): Phenotypic changes of aerial parts during sprouting.

Jerusalem artichoke cultivars QY1 and QY3, both bred from the Qinghai Academy of Agriculture and Forestry Sciences, were harvested and stored at 4 °C and RH 85%. We selected 300 tubers that had not been damaged by external forces and cut off the terminal buds as test material for sprouting tests after 6 months of storage. (Figure 6A).

Each processed tuber weighing 10 g was cultured using hydroponics. The samples were maintained on water-filled Petri dishes with the top buds facing upward, with three tubers per Petri dish, and incubated at 25 °C during the day and 18 °C at night. The light intensity was 1500–1800 lx, and the light duration was 16 h/d. Test materials were sampled every two days after sprouting while measuring the corresponding bud length for that period (Table S3) and recording the bud growth (Figure 6B).

The sampled tubers were washed, sliced, dried to constant weight, ground, and set aside. After harvesting, the buds were immediately frozen in liquid nitrogen and then stored in a −80 °C freezer. There were 9 tubers for three biological replicates for each period of tuber sprouting in the carbohydrate study and randomly 9 tubers for three biological replicates in metabolomic and transcriptome studies for each period of tubers and buds.

### 4.2. Extraction and Determination of Carbohydrates

Extraction of Carbohydrates: The Jerusalem artichoke tubers were sliced and dried to a constant weight in an oven at 80 °C, followed by extraction using the hot water extraction method. Before determining the sugar content by HPLC, the extract was filtered through a 0.22 μm membrane: Reaction of Extract with FEH Enzyme Solution: The enzyme solution was prepared by dissolving it in distilled water and storing it in a refrigerator at 4 °C for later use. After reacting the enzyme solution with the extract, the mixture was analyzed using high-performance liquid chromatography (HPLC). Determination of Fructan Content: The contents of glucose and fructose were determined by HPLC. The fructan content was calculated using the formula: Fructan = k × (Glucose + Fructose) [57], where, k = [180 + 162(n − 1)]/180n, n (average degree of polymerization) = Fructose/Glucose + 1.

Carbohydrate content was determined by high-performance liquid chromatography (HPLC), with a Shimadzu (Kyoto, Japan) LC20A analytical system, LC10A differential refractive index detector, and a Shim Pack SCR-101-C (Kyoto, Japan) (7.9 mm × 30 cm) column for sugar analysis, using pure water as the mobile phase. The sugar standard was obtained from the China Food and Drug Control Institute (Beijing, China) and the Tokyo Chemical Industry (Tokyo, Japan).

### 4.3. Metabolite Analysis Through Ultraperformance Liquid Chromatography-Tandem Mass Spectrometry (UPLC-MS/MS)

Metabolites in tubers and buds were detected using ultra-performance liquid chromatography-tandem mass spectrometry (UPLC-MS/MS). A 100 mg sample of freeze-ground powder was dissolved in 1.2 mL of 70% methanol extraction solution, vortexed, and stored at 4 °C overnight. After centrifugation (12,000 rpm, 10 min), the supernatant was filtered through a 0.22 μm membrane for UPLC-MS/MS analysis. The liquid chromatography conditions were as follows: Agilent SB-C18 (Santa Clara, CA, USA) column (1.8 µm, 2.1 mm × 100 mm), mobile phase A (ultrapure water with 0.1% formic acid) and B (acetonitrile with 0.1% formic acid), gradient elution starting at 5% B at 0.00 min, linearly increasing to 95% B within 9.00 min and holding for 1 min, then decreasing to 5% B at 10.00–11.10 min and equilibrating until 14.00 min, flow rate of 0.35 mL/min, column temperature of 40 °C, and injection volume of 4 μL. The mass spectrometry conditions were: ESI Turbo ion spray interface, source temperature of 550 °C, ion spray voltage of 5500 V (positive ion mode)/−4500 V (negative ion mode), ion source gas 1 (50 psi), gas 2 (60 psi), curtain gas (25 psi), collision-induced ionization set to high, calibration in triple quadrupole (QQQ) mode using 10 μmol/L polypropylene glycol solution, and in LIT mode using 100 μmol/L solution, scanning in multiple reaction monitoring (MRM) mode with collision gas (nitrogen) set to medium, and further optimization of declustering voltage (DP) and collision energy (CE) for each MRM ion pair. Data analysis was based on the MWDB database, with qualitative analysis using secondary spectral information, quantitative analysis using MRM mode, and chromatographic peak integration and correction performed using MultiQuant software 3.0. Significant differential metabolites were screened through OPLS-DA analysis, combining variable importance in projection (VIP), *p*-value, and fold change (Fold Change ≥ 2 or ≤0.5, and VIP ≥ 1).

### 4.4. RNA Extraction and Sequencing

Total RNA was extracted from QY1 samples using RNAprep Pure Plant kit (TIANGEN, Beijing, China), analyzed for integrity by agarose gel electrophoresis, and measured for purity using the Nanophotometer spectrophotometer. RNA concentration was measured with high accuracy using the Qubit 2.0 fluorometer (Waltham, MA, USA), and RNA integrity was determined using the Agilent 2100 Bioanalyzer (Santa Clara, CA, USA). After a quality check, the constructed cDNA library was sequenced on the Illumina HiSeq 2500 (San Diego, CA, USA). The clean reads were obtained by filtering the sequencing data and assessing the sequencing error rate and GC content distribution. Because Jerusalem artichoke has no reference genome, clean reads were spliced to obtain reference sequences for subsequent analysis. Transcripts were obtained by Trinity splicing, and the longest cluster sequence was obtained using the closest hierarchical clustering as Unigene for subsequent analysis.

### 4.5. Transcriptomics and Analysis of Differentially Expressed Genes (DEGs)

Unigene sequences were compared with Kyoto Encyclopedia of Genes and Genomes (KEGG), non-redundant (NR), Swiss-Prot, Gene Ontology (GO), Clusters of Eukaryotic Orthologous Groups (KOG), and Trembl databases using the DIAMOND software 2.1.8. After predicting Unigene’s amino acid sequence, Unigene’s annotation information was obtained by comparing it with the Pfam database using HMMER software 3.3. Using bowtie2 of RSEM software 1.3.3, the transcripts after Trinity assembly and redundancy removal were used as the reference sequences, and the clean reads of the samples were compared with the reference sequences. To truly reflect the number of fragments at the transcript expression level, the number of mapped reads and transcript length of the samples were normalized. FPKM (fragments per kilobase of transcript per million mapped reads) was used to indicate transcript or gene expression level.

Differential expression analysis between sample sets was conducted through DESeq2 using unnormalized gene read count data to obtain a set of DEGs between two biological conditions. Among them, the read count of genes is implemented using featureCounts. The false discovery rate (FDR) was obtained using the Benmini–Hochberg method to correct the *p*-values of multiple hypotheses. The DEGs were screened for |log2Fold Change| ≥ 1 and FDR < 0.05. The obtained DEGs were annotated into KEGG, GO, and KOG databases, and hypergeometric tests were applied for enrichment analysis.

### 4.6. Combined Analysis of Metabolome and Transcriptome of Plant Hormones and Carbohydrates

To more comprehensively analyze the interaction relationships between genes and metabolites, differential metabolites and differential genes from the same component were simultaneously annotated into the KEGG pathway map. Through this integrated analysis, the associations between genes and metabolites in metabolic pathways can be visualized more intuitively, thereby providing a deeper understanding of their functions and regulatory mechanisms in biological processes. First, a systematic correlation analysis was conducted on the genes and metabolites detected in each differential group, and the Pearson correlation coefficients between genes and metabolites were calculated using the cor program in R 4.3.1. Results with Pearson correlation coefficients > 0.99 were selected, as these can more accurately reflect the strong associations between genes and metabolites.

To explore the regulatory relationship between plant hormones and fructans, the Pearson correlation coefficients between the expression of phytohormone-related genes and the proportion of carbohydrates were also calculated using the cor program in R. Results with correlation coefficients > 0.99 were selected as significant.

## 5. Conclusions

Two Jerusalem artichoke cultivars (QY1 and QY3) were selected as subjects for sprouting experiments. The results indicated that during the tuber sprouting period, carbohydrates, primarily fructans, played a regulatory role through conversion activities. Analysis of carbohydrates at different stages revealed significant changes in their proportions across three periods (D0, D1, and D4). Therefore, metabolomic and transcriptomic analyses were conducted on QY1 samples from key stages. The results suggested that jasmonic acid and salicylic acid primarily promoted Jerusalem artichoke sprouting through metabolic regulation and participated in the phytohormone signal transduction process. Transcriptomic analysis showed that the ko04075 pathway was significantly enriched during the sprouting process. Finally, phytohormone-related genes were divided into three groups based on expression levels, two of which (excluding abscisic acid) were associated with carbohydrate proportions and showed a higher correlation with fructose. This suggests that phytohormones may regulate carbohydrate metabolism during the sprouting of Jerusalem artichoke tubers. In conclusion, these results preliminarily elucidate the regulatory mechanisms of phytohormones in Jerusalem artichoke tuber sprouting, aiming to provide a theoretical basis for the production and processing of Jerusalem artichoke. Furthermore, this may further contribute to improving the yield of Jerusalem artichoke crops and could potentially alter the fructan composition through the action of phytohormones, which holds significant importance.

## Data Availability

The relevant data of this study have been deposited in the NCBI BioProject database under the accession number PRJNA1057422.

## Supplementary Materials

The following supporting information can be downloaded at https://www.mdpi.com/article/10.3390/ijms26051864/s1.
