# Supplementary material for: Metabolome and Transcriptome Analyses Reveal the Correlation Between Fructan Changes and Phytohormone Regulation During Tuber Sprouting of Helianthus tuberosus L
Source: Int J Mol Sci. 2025 Feb 21;26(5):1864. doi: 10.3390/ijms26051864 (PMC11899686; doi:10.3390/ijms26051864)
Supplement: Supplementary file 1 [file ijms-26-01864-s001.zip › Suppl. captions.pdf]

Figure S1A: PCA; Figure S1B: D0&D1.OPLS; Figure S1C: D0&D4.OPLS; Figure S1D: D1&D4.OPLS;
Figure S2: QC;
Figure S3: Unigene.KOG.classification;
Figure S4: Unigene.GO.classification;
Figure S5A: D1vsD4.KEGG top level.classification; Figure S5B:D0vsD1.KEGG top level. classification;
Figure S5C: D0vsD4.KEGG top level.classification;
Figure S6:Differential gene analysis in tissue of Jerusalem artichoke during sprouting;
Figure S7: The number of differential genes involved in various plant hormone signal transduction pathways;
Figure S8:Jerusalem artichoke phytohormone signal transduction pathway;
Figure S9: Correlation network diagram between jasmonic acid and salicylic acid and differentially expressed genes in the pathway;
Figure S10: Fructan regulatory network in Jerusalem artichoke;
Table S1: KEGG annotation of phytohormone-related metabolites in tissue of Jerusalem artichoke tuber sprouting;
Table S2:Sequencing output statistics;
Table S3: Phenotypic changes in Jerusalem artichoke at the sprouting stage.
