# Supplementary figures and images for: Metabolome and Transcriptome Analyses Reveal the Correlation Between Fructan Changes and Phytohormone Regulation During Tuber Sprouting of Helianthus tuberosus L
Source: Int J Mol Sci. 2025 Feb 21;26(5):1864. doi: 10.3390/ijms26051864 (PMC11899686; doi:10.3390/ijms26051864)

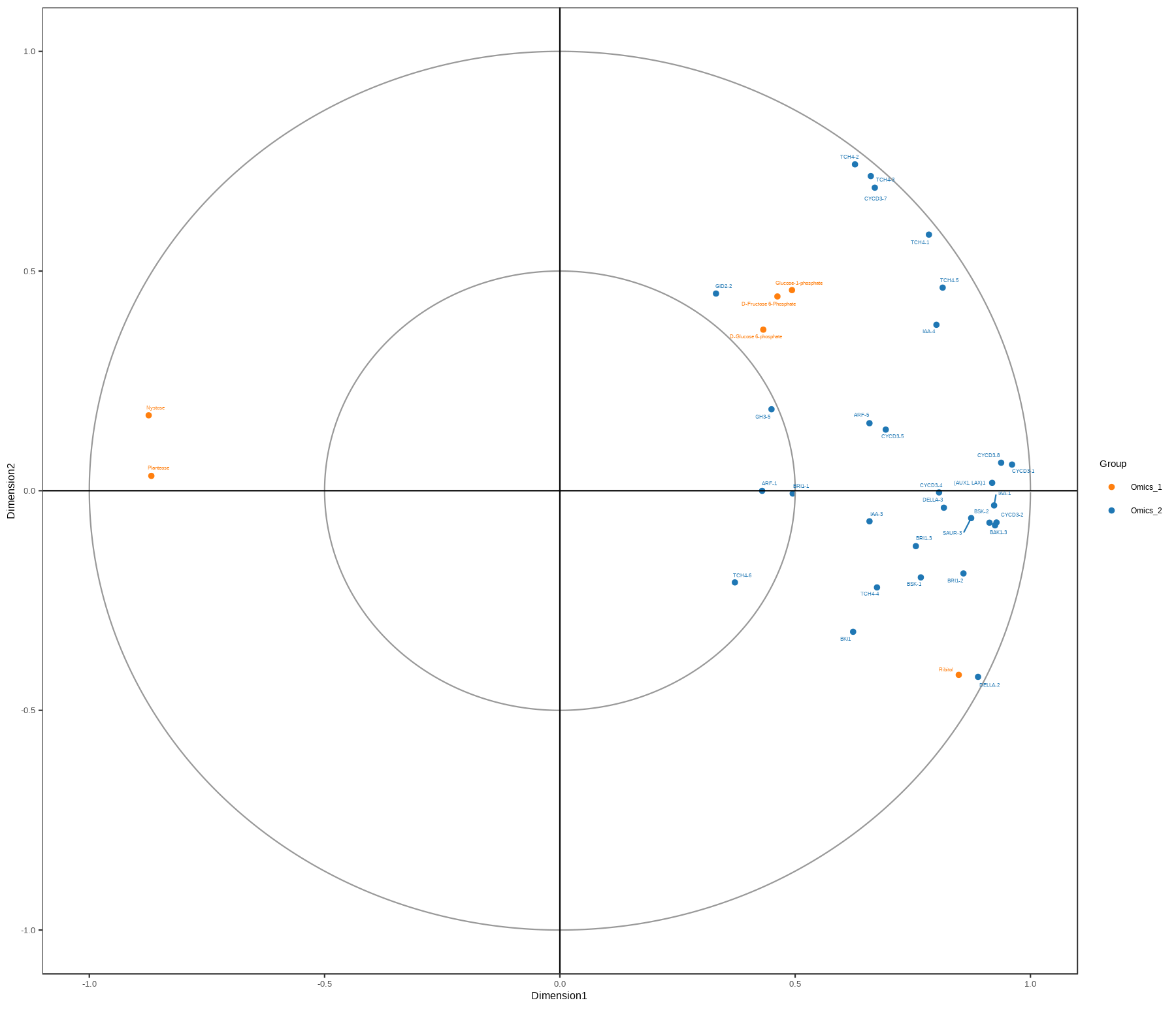

Supplement: Supplementary file 1 [file ijms-26-01864-s001.zip › Fig.S10.png]

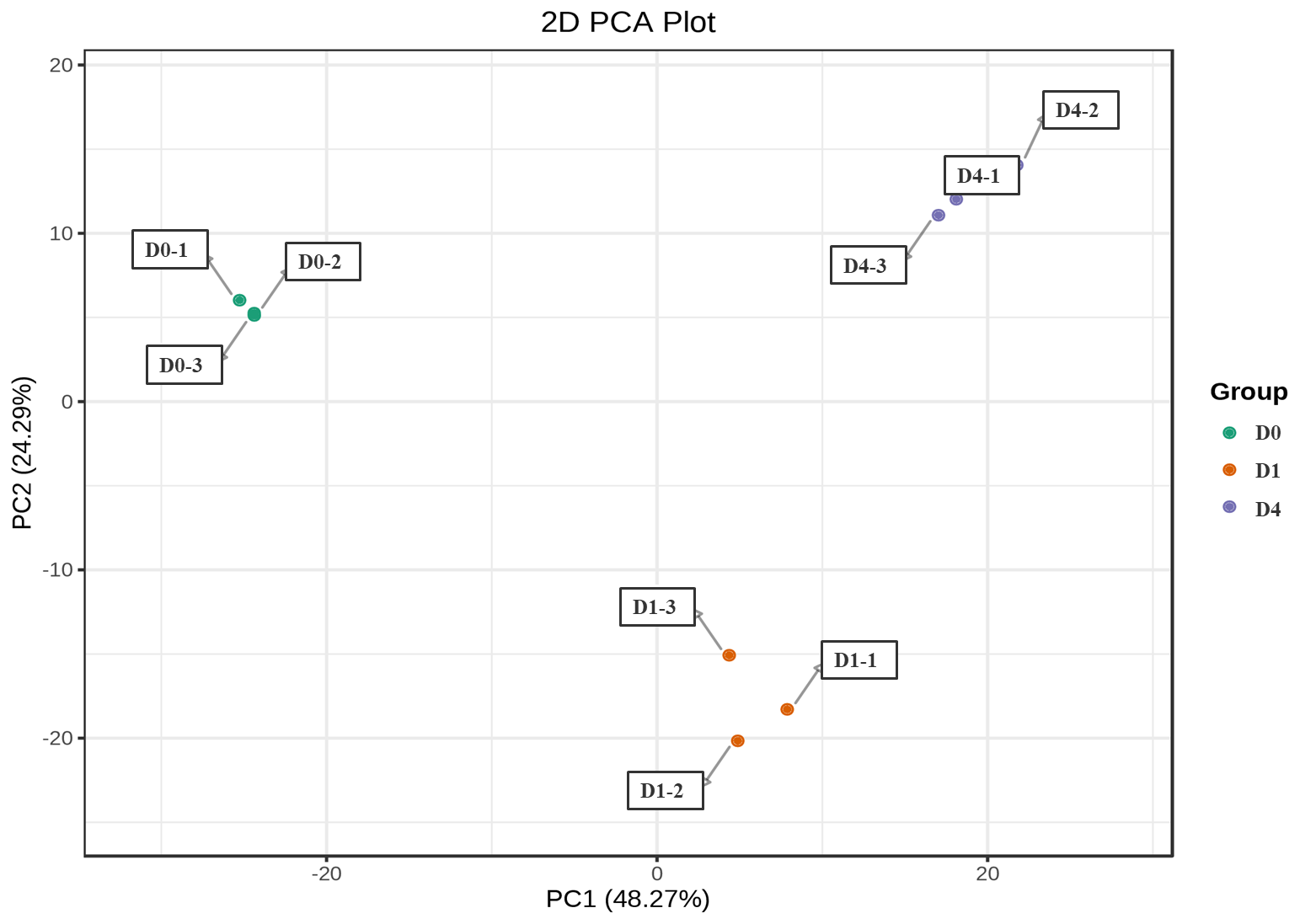

Supplement: Supplementary file 1 [file ijms-26-01864-s001.zip › Fig.S1A.PCA.png]

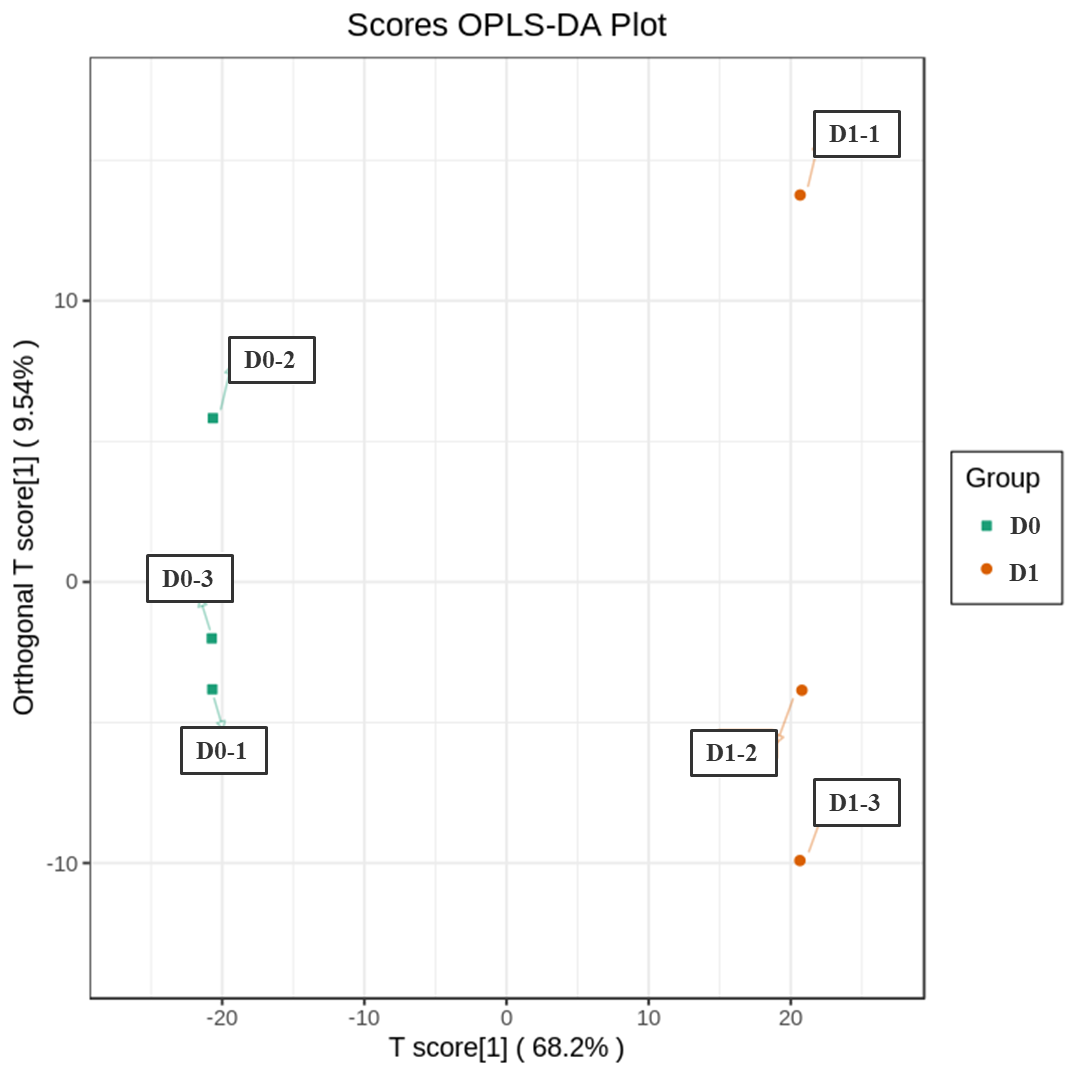

Supplement: Supplementary file 1 [file ijms-26-01864-s001.zip › Fig.S1B.D0&D1.OPLS.png]

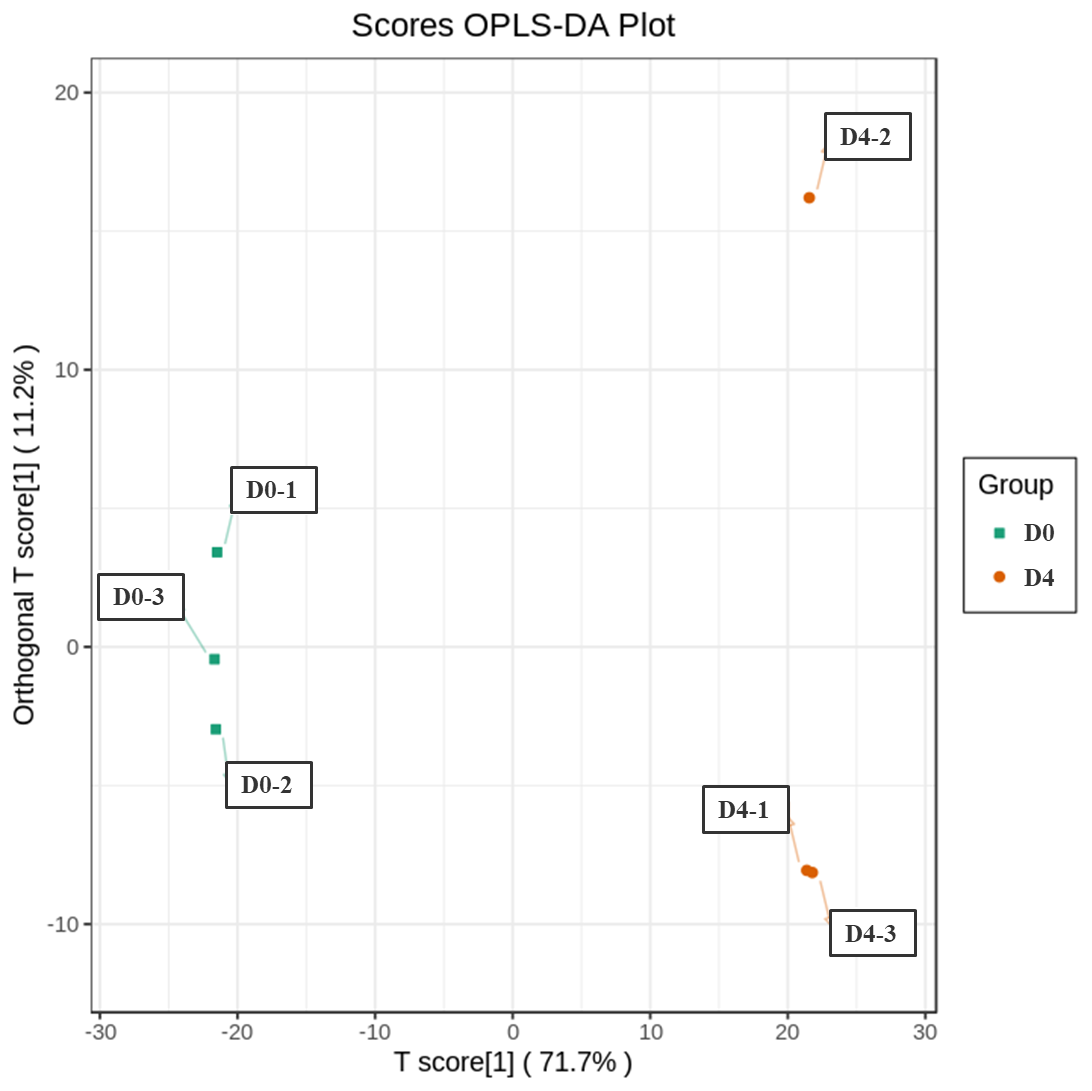

Supplement: Supplementary file 1 [file ijms-26-01864-s001.zip › Fig.S1C.D0&D4.OPLS.png]

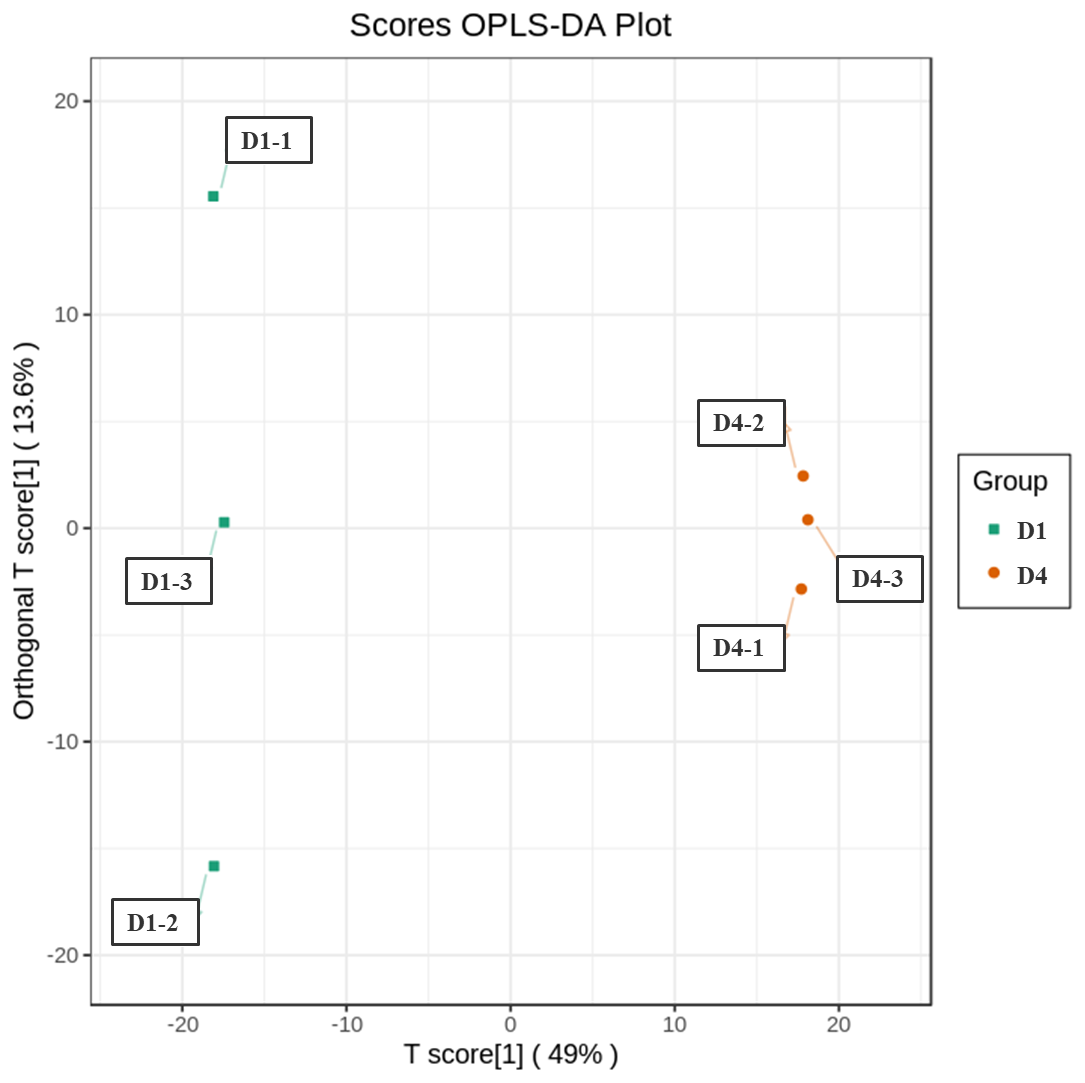

Supplement: Supplementary file 1 [file ijms-26-01864-s001.zip › Fig.S1D.D1&D4.OPLS.png]

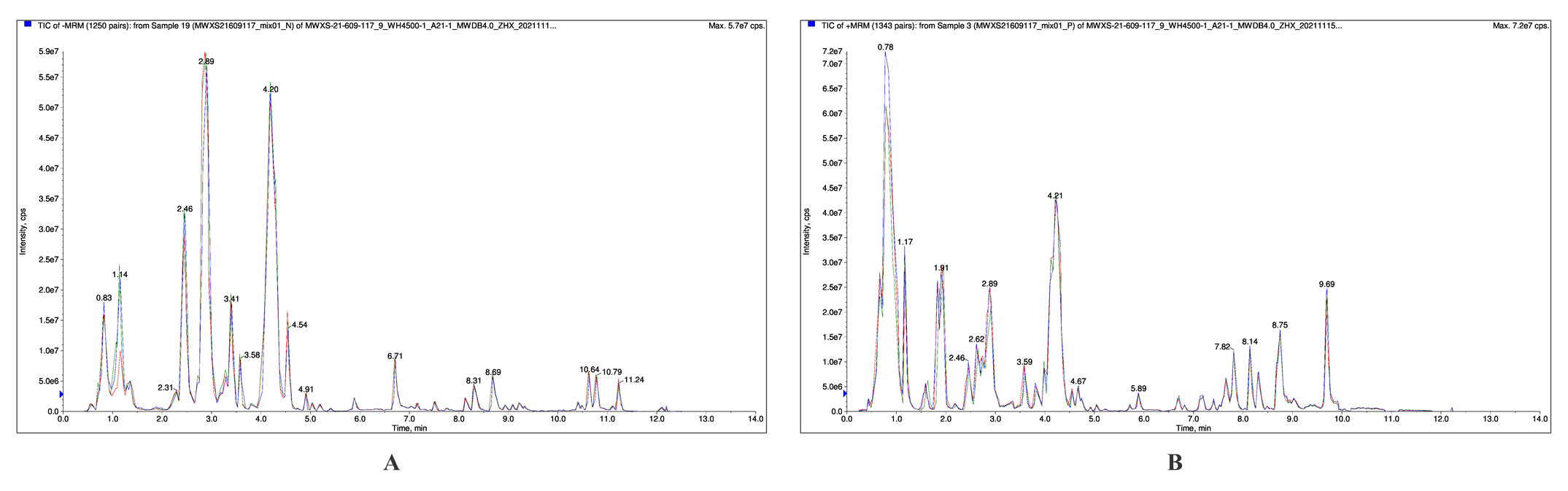

Supplement: Supplementary file 1 [file ijms-26-01864-s001.zip › Fig.S2.QC.png]

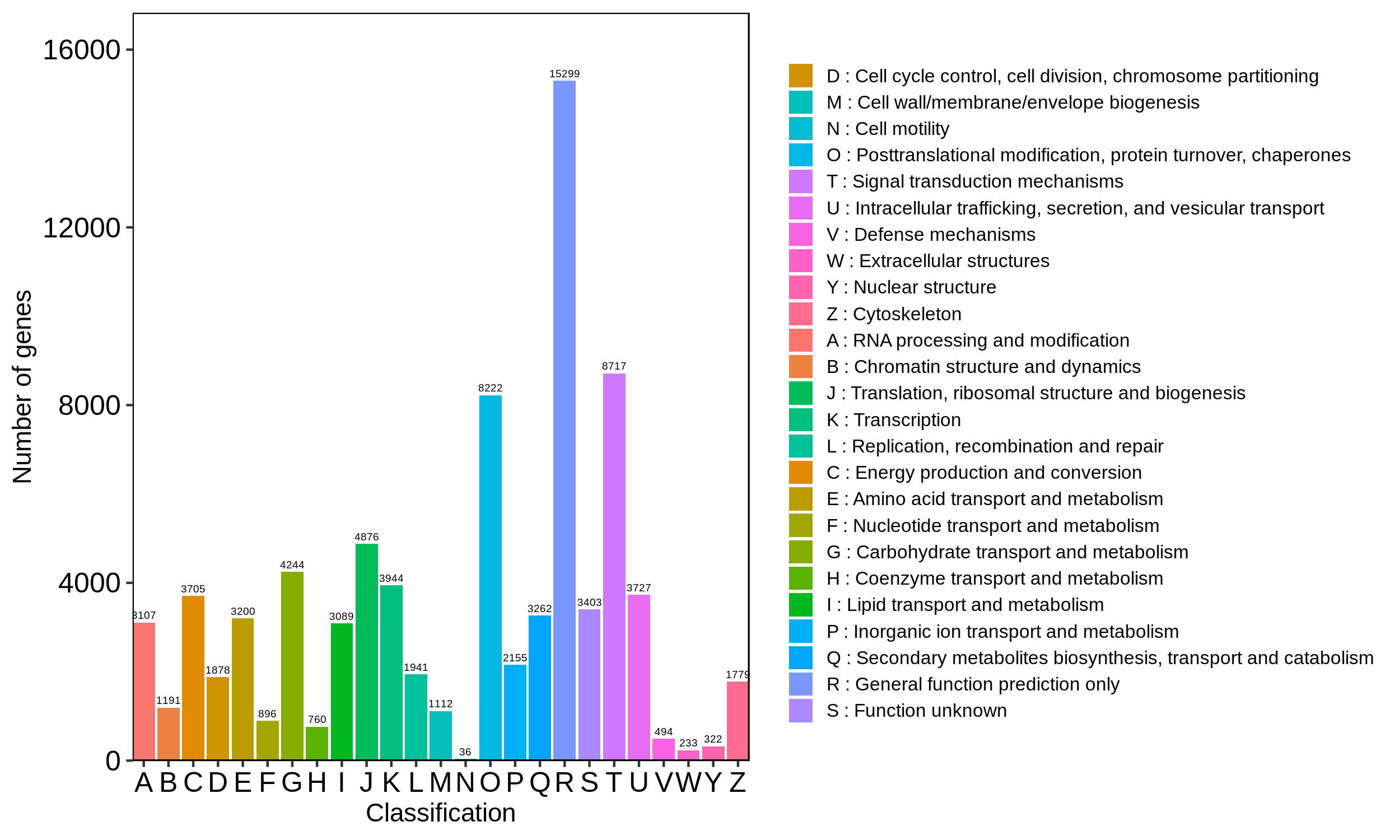

Supplement: Supplementary file 1 [file ijms-26-01864-s001.zip › Fig.S3.Unigene.KOG.classification.png]

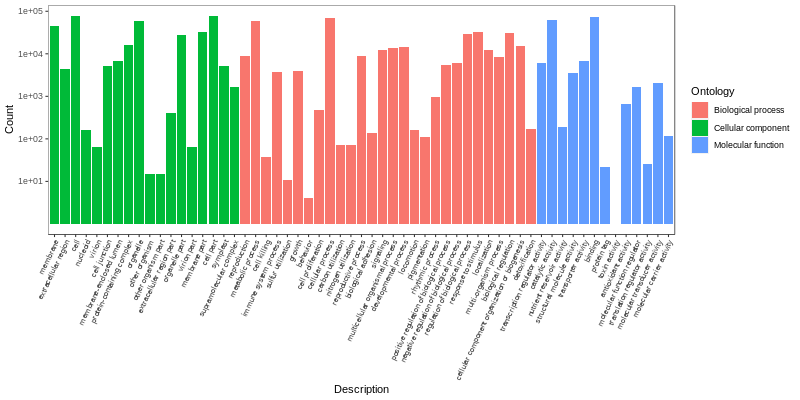

Supplement: Supplementary file 1 [file ijms-26-01864-s001.zip › Fig.S4.Unigene.GO.classification.png]

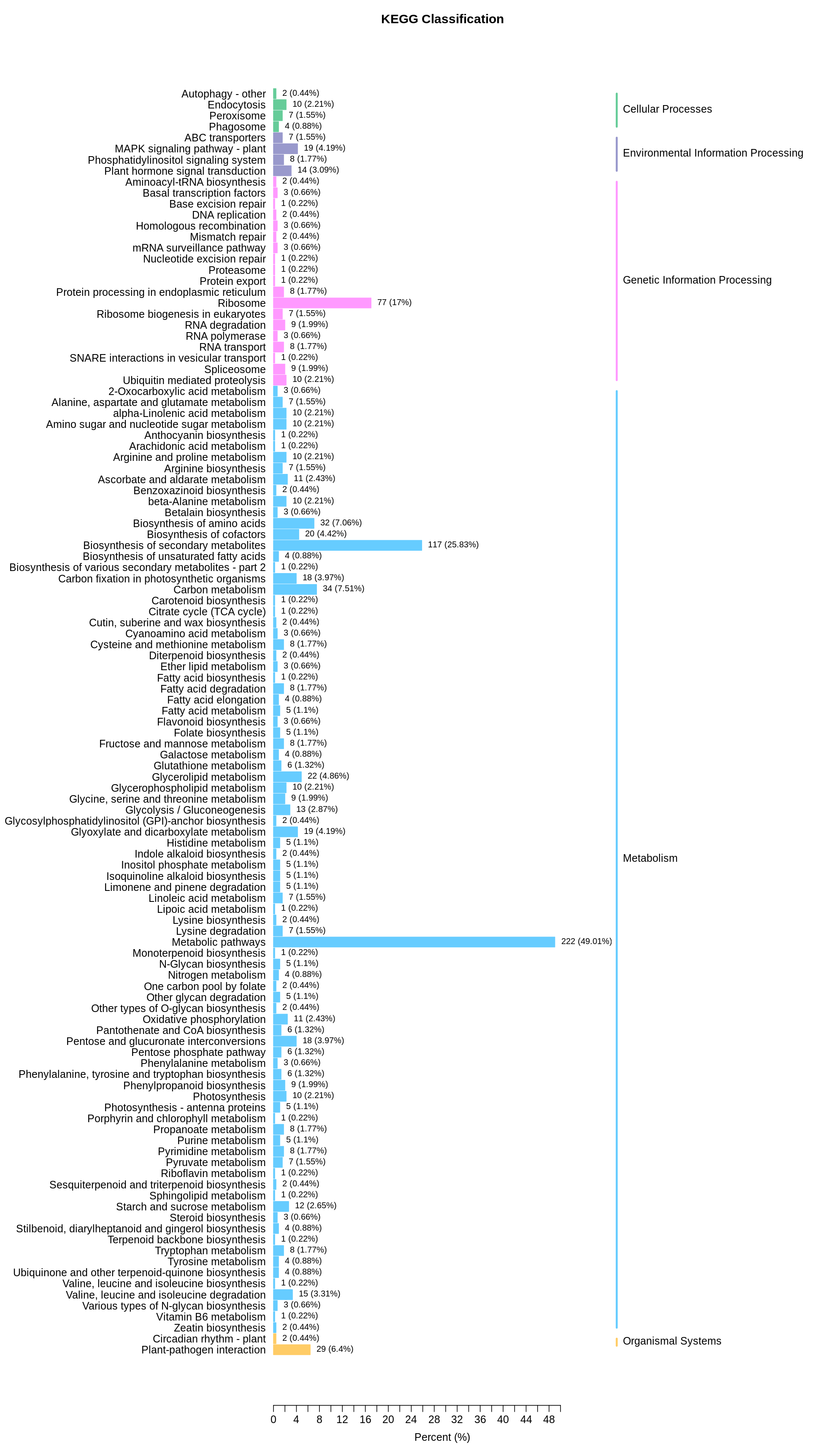

Supplement: Supplementary file 1 [file ijms-26-01864-s001.zip › Fig.S5A .D1vsD4.KeggTopLevel.Classification.png]

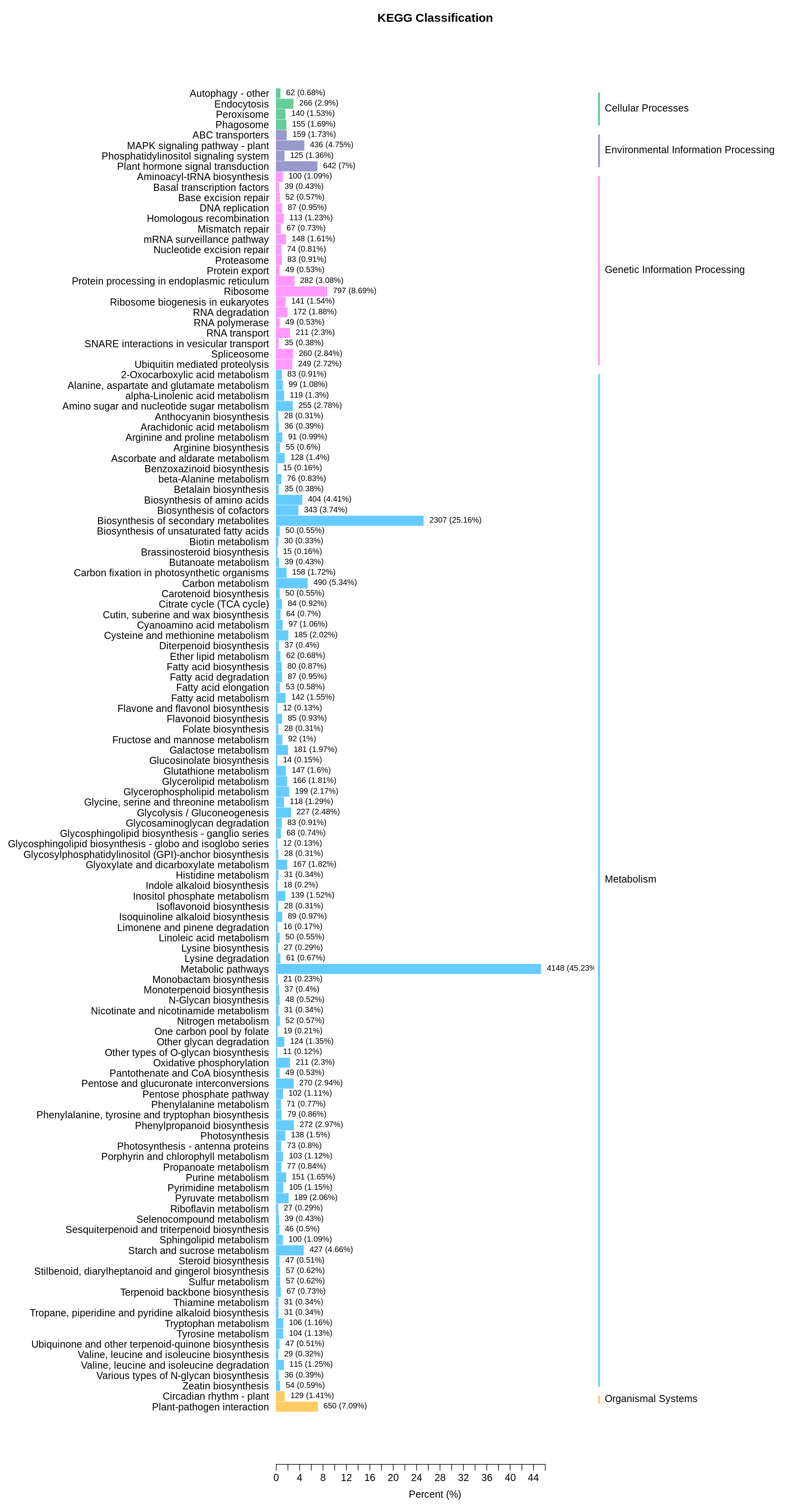

Supplement: Supplementary file 1 [file ijms-26-01864-s001.zip › Fig.S5B.D0vsD4.KeggTopLevel.Classification.png]

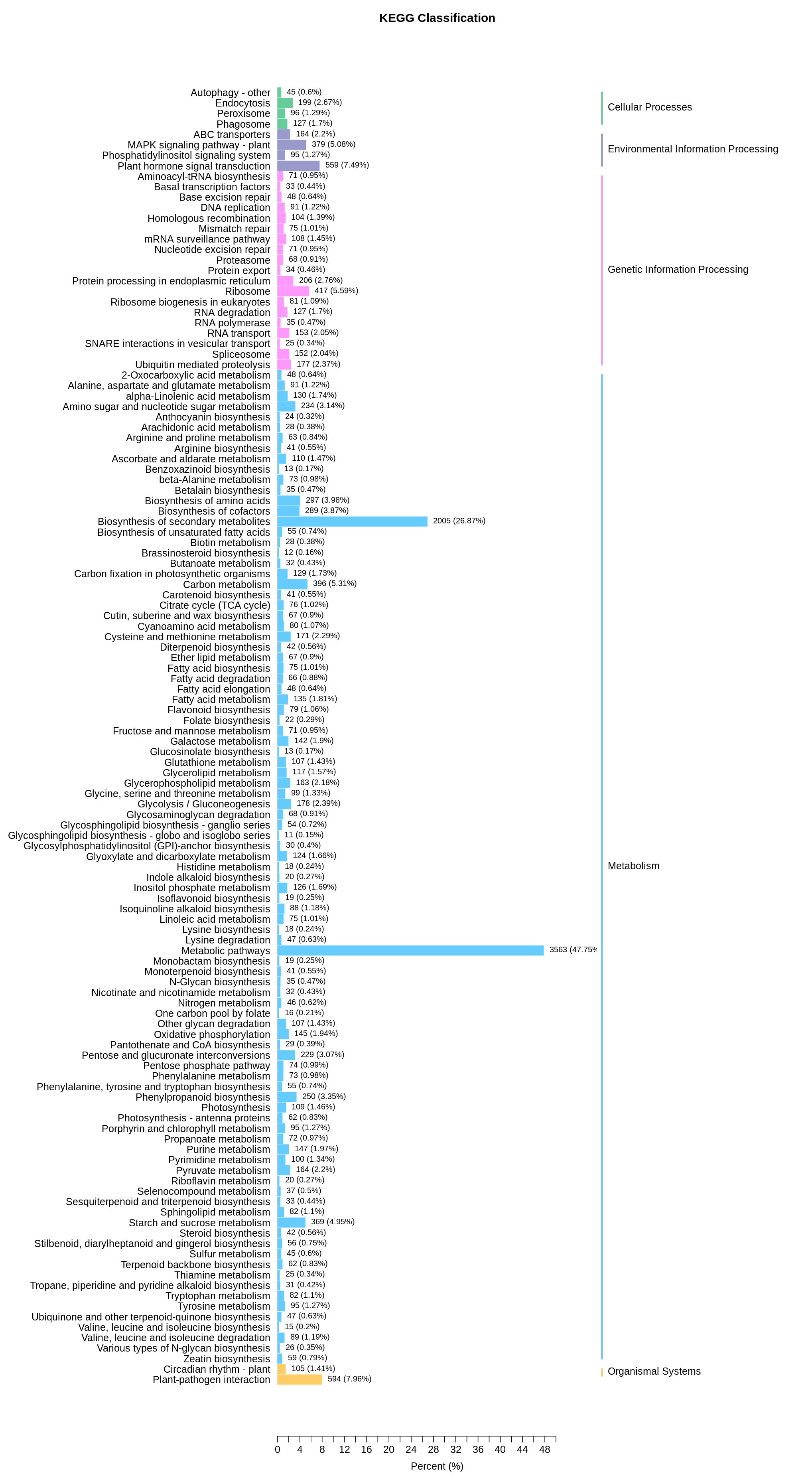

Supplement: Supplementary file 1 [file ijms-26-01864-s001.zip › Fig.S5C.D0vsD1.KeggTopLevel.Classification.png]

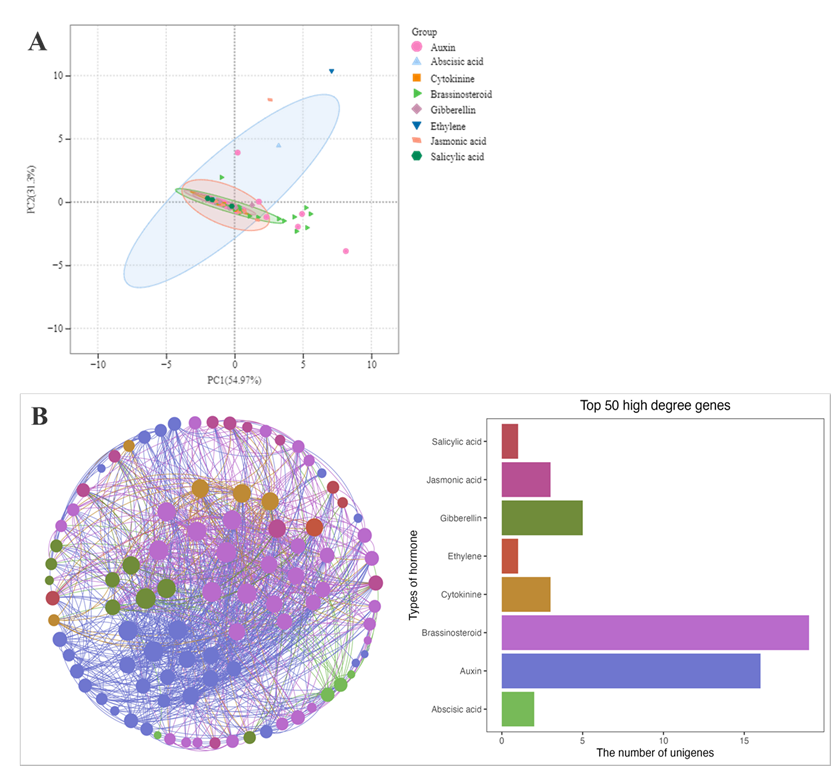

Supplement: Supplementary file 1 [file ijms-26-01864-s001.zip › Fig.S6.png]

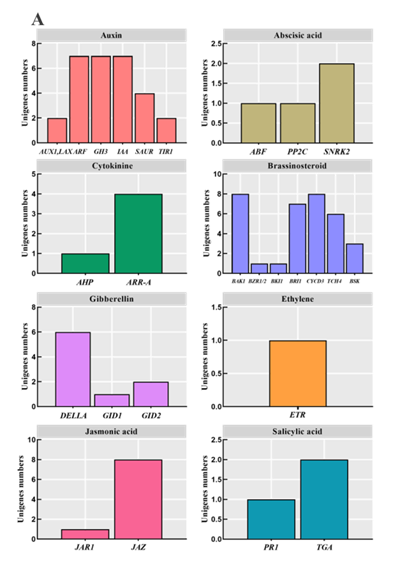

Supplement: Supplementary file 1 [file ijms-26-01864-s001.zip › Fig.S7.png]

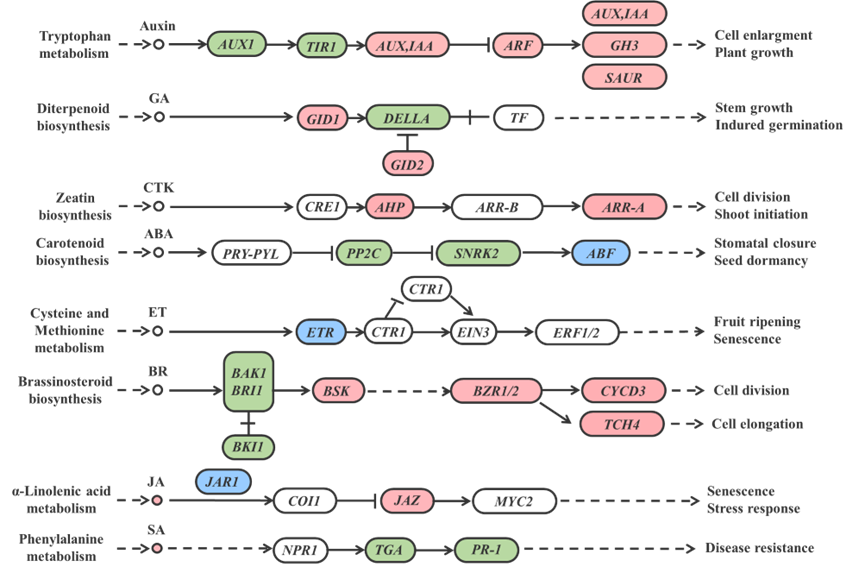

Supplement: Supplementary file 1 [file ijms-26-01864-s001.zip › Fig.S8.png]

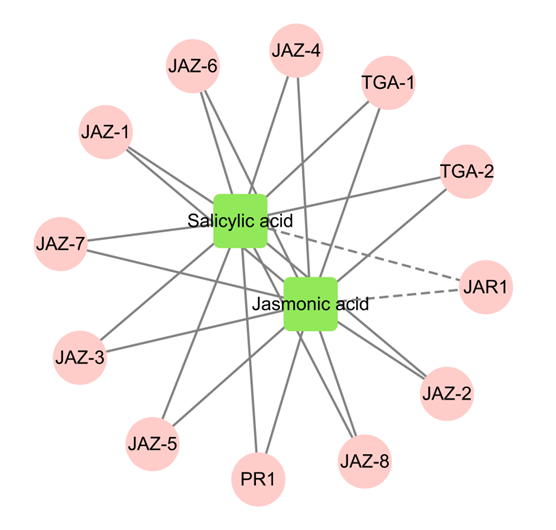

Supplement: Supplementary file 1 [file ijms-26-01864-s001.zip › Fig.S9.png]
